# Single-Thermocouple Suspended Microfluidic Thermal Sensor with Improved Heat Retention for the Development of Multifunctional Biomedical Detection

**DOI:** 10.3390/s25154532

**Published:** 2025-07-22

**Authors:** Lin Qin, Xiasheng Wang, Chenxi Wu, Yuan Ju, Hao Zhang, Xin Cheng, Yuanlin Xia, Cao Xia, Yubo Huang, Zhuqing Wang

**Affiliations:** 1 School of Mechanical Engineering, Sichuan University, Chengdu 610065, China; qinlin19583625790@163.com (L.Q.); 2022141410152@stu.scu.edu.cn (X.W.); 2022141410309@stu.scu.edu.cn (C.W.); ajuyu1@outlook.com (Y.J.); 18997567428@163.com (H.Z.); yuanlin.xia@scu.edu.cn (Y.X.); xiacao_30@163.com (C.X.); wzhuqing@scu.edu.cn (Z.W.); 2 Med+X Center for Manufacturing, West China Hospital, Sichuan University, Chengdu 610041, China; 3 Graduate School of International Cultural Studies, Tohoku University, Sendai 980-5877, Japan; cheng.xin.t2@dc.tohoku.ac.jp

**Keywords:** suspended bridge structure, MEMS technique, thermal sensor, heat loss, thermal conduction

## Abstract

Thermal sensors are widely used in medical, industrial and other fields, where the requirements for high sensitivity and portability continues to increase. Here we propose a suspended bridge structure fabricated using MEMS, which effectively shrinks the size and reduces heat loss. This study reviews current sensor-related theories of heat conduction, convective heat transfer and thermal radiation. Heat loss models for suspended and non-suspended bridge structures are established, and finite element analysis is conducted to evaluate their thermal performance. The thermal performance of the suspended bridge structure is further validated through infrared temperature measurements on the manufactured sensor device. Theoretical calculations demonstrate that the proposed suspension bridge structure reduces heat loss by 88.64% compared with traditional designs. Benefiting from this improved heat retention, which was also confirmed by infrared thermography, the thermal sensor fabricated based on the suspension bridge structure achieves an ultra-high sensitivity of 0.38 V/W and a fast response time of less than 200 ms, indicating a high accuracy in thermal characterization. The correlation coefficient obtained for the sensor output voltage and input power of the sensor is approximately 1.0. Based on this design, multiple microfluidic channels with suspended bridge structures can be integrated to realize multi-component detection, which is important for the development of multifunctional biomedical detection.

## 1. Introduction

A thermocouple biosensor is essentially a calorimeter that detects the concentration of a target substance in test solution through thermal sensing [1,2,3,4]. The thermocouple is an important temperature-sensing component in the sensor, capable of detecting the heat changes generated by biochemical reactions in the heat solution. The sensor detects the target content by measuring the electrical signal induced by the heat change. Due to its stable operation [5,6,7], simple structure and convenient operation, it has been widely used in clinical medicine, environmental monitoring and other fields [8,9,10].

In recent years, microfluidic detection chips have shown great advantages [11,12,13,14,15] in the field of medical analysis due to their high detection sensitivity, fast response time and flow integrability [16,17,18]. Therefore, integrating thermal biosensors with microfluidic chips is considered a promising approach to achieve miniaturization, high integration and enhanced sensitivity [19,20,21,22,23]. Reaction heat is a key factor influencing the detection performance of thermocouple biosensors. However, the presence of a temperature difference can induce heat transfer and result in heat loss, which may limit the accuracy of biosensor detection [24,25]. This issue is particularly prominent in most conventional biosensors, where PDMS is directly applied to the substrate for microfluidic processing. When there is no forced convection and the center temperature of the reaction chamber is below 500 degrees Celsius, the heat loss due to thermal convection and thermal radiation is minimal compared with thermal conduction. Since solids have significantly higher thermal conductivity than gases, the direct contact between the microfluidic channel and the substrate in conventional biosensors leads to a large amount of heat dissipation through solid conduction. Therefore, an excellent microfluidic structure design is urgently required for current thermal biosensors to minimize heat loss and enhance both sensitivity and accuracy.

Recent advancements in micro-electro-mechanical systems thermal sensors have focused on mitigating parasitic heat loss through innovative structural designs and the integration of low-thermal conductivity materials, a trend highlighted in recent reviews [26]. A predominant strategy has been the creation of suspended structures, whose excellent thermal performance has been widely demonstrated in recent microsensor research [27,28,29,30]. The key principle is that the insulation effect of an air cavity significantly reduces heat loss compared with a solid substrate, a concept validated in studies of multilayer structures by researchers like Wen-Yang Chang et al. [31]. Pioneering work by Cerda Belmonte et al. on suspended micro-hotplates already showed their potential for achieving fast thermal response and lower power consumption [32,33]. Building on this foundation, more recent developments include perforated-membrane microheaters, which reduce conductive losses by approximately 35%, and γ-Fe_2_O_3_-based propane detectors on suspended micro-hotplates, requiring only 23 mW to maintain 360 °C [34]. Complementing these geometric approaches, material and fabrication innovations, such as the use of plasma-bonded SU-8, have enabled mechanically robust channels with ultralow thermal conductivity. Collectively, these developments have established performance benchmarks for high-efficiency thermal sensors, including operating power below 100 mW and response times under 200 ms. Building upon this foundation, our work introduces an SU-8 suspended bridge structure that significantly advances these metrics. By eliminating 88.638% of heat loss compared with conventional designs, our sensor achieves a superior sensitivity of 0.38 V/W and a rapid response time of less than 200 ms, thereby setting a new standard for thermal management and detection accuracy in microfluidic biosensors.

Based on this, we designed and fabricated a single-channel biosensor with a suspended bridge microfluidic structure using MEMS technology and evaluated its thermal performance. The results demonstrated that the suspended bridge structure effectively reduces heat loss and improves detection accuracy. Thermal imaging experiments confirmed the feasibility of this structure in minimizing heat loss. In addition, integrating multiple suspended microfluidic channels holds great promise for multi-target detection applications.

## 2. Design and Evaluation of Thermal Sensor Devices

### 2.1. Design of Thermal Sensor Devices

**Figure 1:**
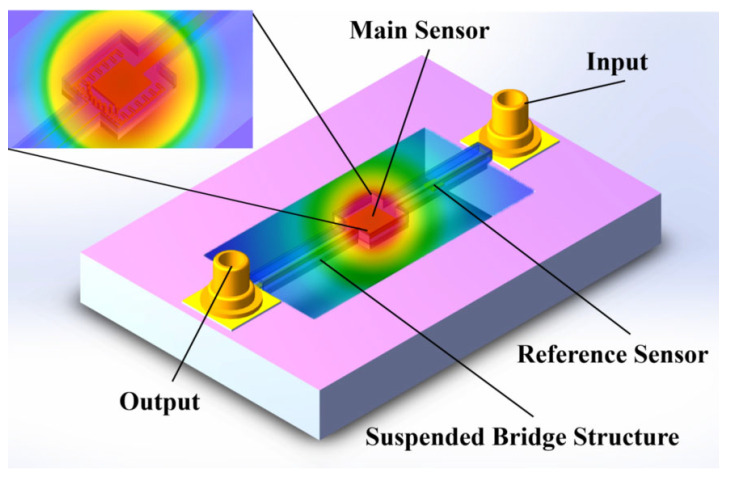
Miniature thermal sensor with suspension bridge structure and microfluidic channel.

A thermocouple biosensor with a suspended bridge structure designed in this study is shown in Figure 1. The substrate of the sensor is composed of silicon, which has a high Seebeck coefficient. PDMS is commonly used in traditional microfluidic lithography due to its excellent elasticity. However, its high elasticity can lead to the collapse of hollow membrane structures, making it difficult to provide sufficient mechanical support for microfluidic chips. Therefore, PDMS is not suitable for constructing thermocouple sensors with suspended structures. Nowadays, microfluidic research is increasingly turning to alternative materials. SU-8 is characterized by high thermal stability and low thermal conductivity, making it suitable for thermal insulation applications. Thermal stability refers to the ability of a material to resist decomposition or other types of chemical reactions during heating. Low thermal conductivity indicates strong thermal insulation capability, which limits heat dissipation and facilitates heat concentration. Due to the flexible preparation, excellent mechanical properties, and the ability to accurately build microstructures, SU-8 was selected as the preferred material for fabricating the microfluidic channel. The center of the microfluidic channel is the reaction chamber, where the biochemical reactions of the detection fluid occur and the hot junction of the thermocouple is located. Therefore, the central region of the substrate was designed with a hollowed structure to effectively prevent direct heat conduction between the reaction chamber and the substrate through the solid medium. This structural feature significantly reduces heat loss and enhances the detection accuracy and sensitivity of the sensor.

### 2.2. Theoretical Derivation and Evaluation

In order to preliminarily evaluate the effectiveness of this structure in reducing heat loss [35], we performed a theoretical derivation of the heat loss of the suspended bridge microfluidic channel and completed a comparative analysis of the two by introducing a heat loss model of the conventional microfluidic channel.

Heat loss refers to the energy that is not detected by the sensor but emitted to the outside, and the suspended bridge microfluidic channel is theoretically categorized into three forms: heat conduction, heat convection and heat radiation.

Heat conduction is the heat transfer phenomenon when there is no macroscopic motion inside the medium. It can occur in solids, liquids and gases. The basic equations of heat conduction refer to (1).

(1) $$\frac{\partial u}{\partial t}=div(Uu)=k(\frac{\partial^{2}u}{\partial x^{2}}+\frac{\partial^{2}u}{\partial y^{2}}+\frac{\partial^{2}u}{\partial z^{2}})=k(u_{xx}+u_{yy}+u_{zz})$$

where div represents the divergence operator; u(t,x,y,z) represents the temperature at time t and spatial position (x,y,z); $\frac{\partial u}{\partial t}$ is the rate of change of temperature of a point in space with respect to time; $u_{xx}$, $u_{yy}$, $u_{zz}$ are the second partial derivative of temperature with respect to the three spatial coordinate axes; and k is the thermal diffusivity, which is determined by the thermal conductivity, density and heat capacity of the material.

Thermal convection is a mode of heat transfer that occurs through the bulk movement of fluids. The heat flux (q) due to the flow of a fluid from an initial temperature t1 to a final temperature t2 is given by Equation (2):

(2) $$q=mc_{p}(t_{2}-t_{1})$$

where: q is the heat flux, representing the rate of thermal energy transferred per unit area, and the unit is W/m^2^; m is the mass flux, representing the mass of fluid flowing per unit area per unit time, and the unit is kg/(s·m^2^); $c_{p}$ is the specific heat capacity of the fluid at constant pressure; and $t_{1}$ and $t_{2}$ are the initial and final temperatures of the fluid, respectively.

Thermal radiation is the emission of energy as electromagnetic waves by an object due to its temperature. This process is described by the Stefan–Boltzmann law, as shown in Equation (3).

(3) $$Q=\varepsilon \sigma A(T^{4}-{T_{0}}^{4})$$

where Q is the energy (heat flux) radiated per unit area; ε is the emissivity; σ is the Stefan–Boltzmann constant, which is about 5.67 × 10^−8^ W m^−2^ K^−4^; A is the surface area of the radiant body; T is the temperature of the radiant body; and T_0_ is the temperature of the environment.

The total heat loss can be modeled from the above three heat transfer methods (4) [36].

(4) $$Q_{total}=Q_{conduction}+Q_{\mathrm{ambient}}+Q_{radiation}+x$$

where $Q_{total}$ is the total heat loss, $Q_{conduction}$ denotes heat transfer through the chamber or through the suspension bridge, $Q_{ambient}$ denotes heat transfer through the air, $Q_{radiation}$ denotes heat loss through thermal radiation, and x denotes unknown heat loss, including natural convection.

**Figure 2:**
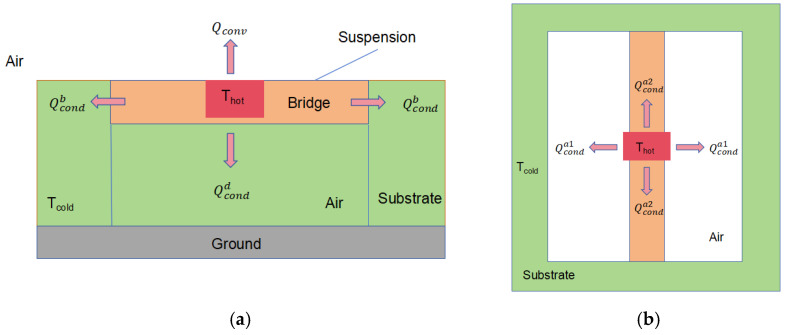
Thermal dissipation model of the microfluidic channel of the suspension bridge: (**a**) front view of thermal dissipation; (**b**) top view of thermal dissipation.

The heat dissipation diagram is shown in Figure 2.

At steady state, the heat transfer within the chamber to the ends via the suspension bridge is calculated with reference to Equation (5).

(5) $$Q_{cond}^{b}=\frac{2K_{b}A_{b}(T_{hot}-T_{cold})}{L}$$

where K_b_ is the thermal conductivity of the suspended bridge structure; A_b_ is the cross-sectional area of the bridge; T_hot_ is the heat source which is set to 30 degrees; T_cold_ is the room temperature which is set to 20 degrees; L is the length from the center of the bridge to the substrate at one end; and the substrate material is Si.

At room temperature, the thermal conductivity of air is 0.025 W/m·K and the natural convection coefficient of air is 10 W/m^2^·K. Since the distance between the outer wall of the chamber and the bottom of the chamber and the substrate is very small, the heat transfer to the ground and the plane direction, $Q_{\mathrm{cond}}^{a1}$ and $Q_{\mathrm{cond}}^{a2}$, is greater than that to the upper part of the chamber, $Q_{\mathrm{conv}}^{d}$. Refer to Equations (6)–(8).

(6) $$Q_{\mathrm{cond}}^{a1}=\frac{2(T_{hot}-T_{cold})}{\frac{\delta_{1}}{K_{s}A_{a1}}+\frac{\delta_{2}}{K_{air}A_{a1}}}$$

(7) $$Q_{\mathrm{cond}}^{a2}=\frac{2\;(T_{hot}-T_{cold})}{\frac{\delta_{1}}{K_{s}A_{a2}}+\frac{\delta_{3}}{K_{air}A_{a2}}}$$

(8) $$Q_{\mathrm{conv}}^{d}=\frac{T_{hot}-T_{cold}}{\frac{\delta_{1}}{K_{s}A_{d}}+\frac{\delta_{4}}{K_{air}A_{d}}}$$

where δ_1_ is the thickness of the cavity; δ_2_, δ_3_ denote the distance from the surface of the cavity to the inner wall of the substrate in the top view; δ_4_ is the distance from the surface of the cavity to the ground; K_s_ is the thermal conductivity of the cavity, and the same material is used in this structure as the suspension bridge; K_air_ is the thermal conductivity of the air; A_a1_, A_a2_ denote the surface area of the cavity in a horizontal direction; and A_d_ denotes the surface area of the bottom of the cavity.

For suspended microfluidic channels, which are directly exposed to air and have a significant difference in temperature from the ambient temperature, the issue of natural convection still needs to be taken into account, cf. Equation (9).

(9) $$Q_{conv}=Ah_{f}(T_{hot}-T_{cold})$$

where A represents the heating area of the cavity in the air; and h_f_ is the natural convection coefficient of air at room temperature.

Of the three forms of heat dissipation, thermal radiation accounts for a relatively small percentage. K.J, Kim, in analyzing the heat transfer relationship between microheaters with suspended structures and air, found that at the tiny scale, when the device is heated above 700 K, the heat loss due to thermal radiation is about 1% of the total heat loss from the device.

Refer to the calculation of Equation (10).

(10) $$Q_{rad}=A_{h}\sigma \varepsilon (T_{hot}^{4}-T_{cold}^{4})$$

where A_h_ is the heat-generating surface area; σ is the Stefan–Boltzmann constant; and ε is the thermal emissivity of SU-8, which is 0.25 [37].

**Figure 3:**
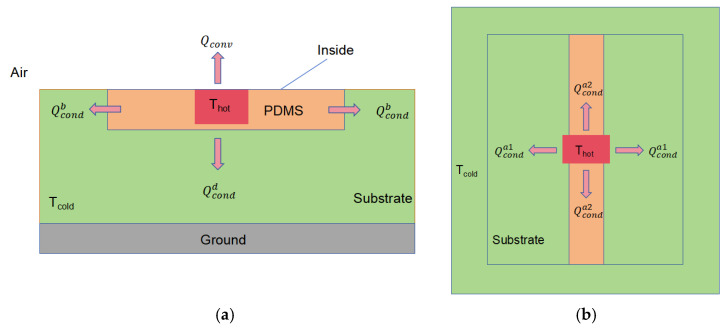
Conventional microfluidic channel heat dissipation model: (**a**) front view of heat dissipation; (**b**) top view of heat dissipation.

In order to initially qualitatively analyze that the structure can effectively reduce the heat loss, a microfluidic heat model without a suspension bridge structure is then established for comparative analysis. The difference between this model and the microfluidic model with a suspension structure is that the cavity in this model is composed of PDMS material, and the cavity is in direct contact with the substrate. The heat dissipation diagram is shown in Figure 3.

The principle of heat loss calculation for this conventional structure is basically the same as above, and the equations are given in (11)–(16).

(11) $$Q_{\mathrm{conv}}^{d}=\frac{T_{hot}-T_{cold}}{\frac{\delta_{1}}{K_{P}A_{d}}+\frac{\delta_{4}}{K_{Si}A_{d}}}$$

(12) $$Q_{\mathrm{cond}}^{a1}=\frac{2(T_{hot}-T_{cold})}{\frac{\delta_{1}}{K_{P}A_{a1}}+\frac{\delta_{2}}{K_{Si}A_{a1}}}$$

(13) $$Q_{\mathrm{cond}}^{a2}=\frac{2\;(T_{hot}-T_{cold})}{\frac{\delta_{1}}{K_{P}A_{a2}}+\frac{\delta_{3}}{K_{Si}A_{a2}}}$$

(14) $$Q_{\mathrm{conv}}^{d}=\frac{T_{hot}-T_{cold}}{\frac{\delta_{1}}{K_{P}A_{d}}+\frac{\delta_{4}}{K_{Si}A_{d}}}$$

(15) $$Q_{conv}=Ah_{f}(T_{hot}-T_{cold})$$

(16) $$Q_{rad}=A_{h}\sigma \varepsilon (T_{hot}^{4}-T_{cold}^{4})$$

where K_b_ is the thermal conductivity of the thin wall of the microfluidic channel in contact with the substrate; A_b_ is the cross-sectional area of the thin wall; K_p_ is the thermal conductivity of the PDMS microfluidic channel; K_Si_ is the thermal conductivity of the Si substrate; and ε is the thermal emissivity of PDMS, which is 0.9 [38].

Based on the equation above, the calculation shows that the heat loss of the suspended microfluidic structure is reduced by 88.64% compared with the conventional microfluidic structure.

To visualize the heat loss reduction effect of the suspended microfluidic channel, we conducted thermal behavior simulations. A conventional microfluidic channel was used as the control, with identical dimensions to the suspended structure except for the base support. The thermal conductivity in both models was set according to the materials used in their respective microfluidic channels. To ensure sufficient comparison, the center hollow of the suspension structure was filled with air. The temperature at the center of the reaction chamber was set to 30 °C to simulate the temperature at the center of the chamber during the measurement.

**Figure 4:**
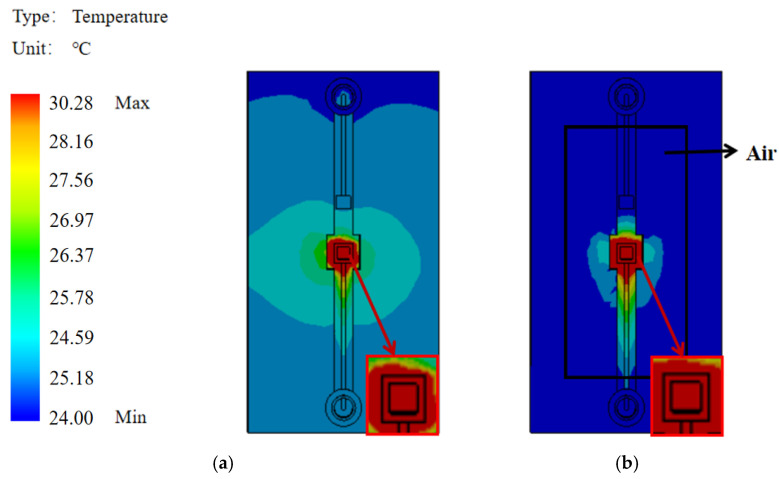
Steady-state thermal simulation of suspended microfluidic channel compared to conventional microfluidic channel: (**a**) steady-state thermal simulation of conventional structure; (**b**) Sseady-state thermal simulation of suspended microfluidic channel structure.

Figure 4a shows that the conventional microfluidic channel is in direct contact with the substrate, causing part of the substrate to be affected by heat conduction, with temperatures rising to between 24.589 °C and 26.967 °C (significant digits are accurate to three decimal places). A significant temperature gradient is observed within the reaction chamber. In contrast, as shown in Figure 4b, in the suspended microfluidic structure, the regions outside the microfluidic channel are almost unaffected by heat, and a wide area of thermal concentration is formed within the reaction chamber. These results from the steady-state thermal analysis indicate that the suspended structure significantly reduces heat loss compared with the conventional design and provides better thermal concentration performance in the reaction chamber.

## 3. Thermal Distribution Simulation and Fabrication of the Sensor Device

### 3.1. Model of the Device

**Figure 5:**
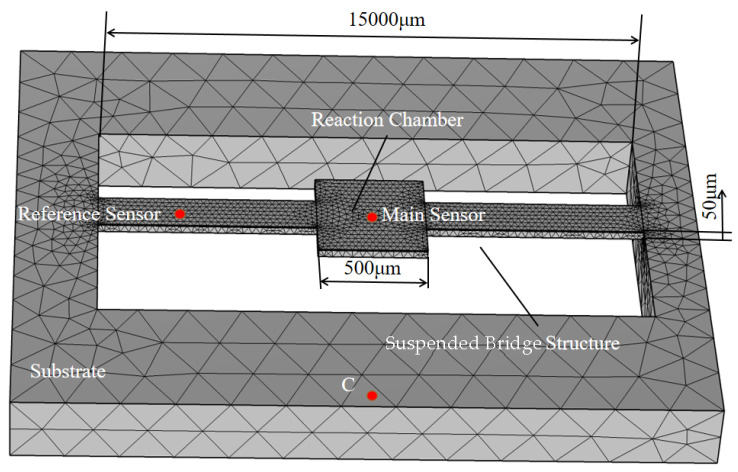
Model of MEMS thermal sensors with suspension bridge structures.

To further validate the thermal performance and structural feasibility of the proposed sensor design, a finite element analysis (FEA) was conducted. Figure 5 shows the FEA model of the MEMS thermal sensor with a suspended bridge structure.

The model exhibits qualitative symmetry in its geometric structure. The microfluidic channels on both sides of the central chamber are identical in size and constructed on a suspended bridge substrate. The central chamber accommodates the reactant sample and provides a site for the chemical reaction. The dimension of the central chamber is 500 μm × 500 μm × 5 μm, and the microfluidic channels are 15,000 μm × 200 μm × 5 μm. The substrate temperature is set at air temperature (24 °C).

To evaluate the exothermic response within the chamber, the heater is located inside the chamber. The temperature inside the microfluidic channel changes in response to variations in the chamber temperature. The heat is mainly transferred within the central chamber and the microfluidic channel.

### 3.2. Thermal Distribution of the Device

Heat loss is the portion of thermal energy that dissipates to the environment without being detected. It includes three forms of heat conduction, heat convection and heat radiation. The thermal sensor designed for this study mainly detects the heat released from the solution reaction. Therefore, minimizing heat loss is essential for improving the sensor’s detection accuracy. To investigate the influence of the suspended bridge structure on temperature measurement, its heat loss characteristics were evaluated [39].

**Figure 6:**
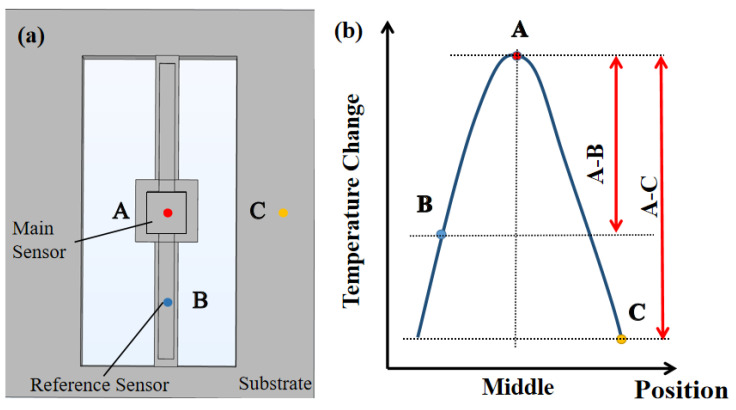
Thermal distribution for the miniature thermal sensor. (**a**) Schematic of the sensor identifying measurement points: A represents the main sensor, B represents the reference sensor and C represents the substrate. (**b**) Simulated temperature profile along the sensor, demonstrating effective thermal isolation.

Figure 6a exhibits a model of a suspended bridge structure. Points A, B and C are located in the central chamber, the microfluidic channel and the foundation, respectively. Figure 6b shows a schematic diagram of the heat loss performance verification. The thermal performance of the suspended bridge structure is evaluated by comparing the temperature difference between point A and point B with the temperature difference between point A and the equipment temperature (room temperature point C). A greater difference between the temperature gradients of A–B and A–C proves that the temperature is mainly concentrated in the central chamber with excellent insulation of the suspended bridge structure.

**Figure 7:**
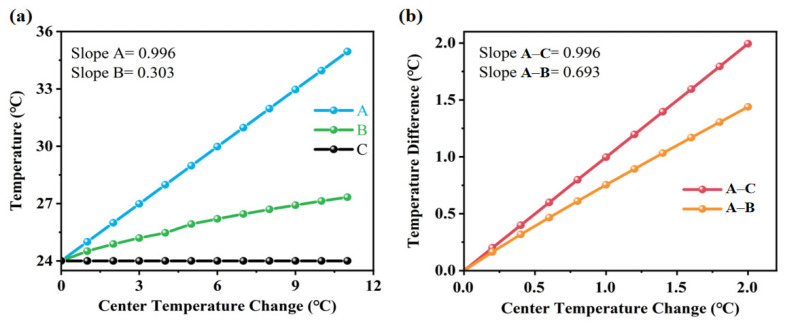
(**a**) Temperature changes of A, B and C under various center temperatures; (**b**) temperature difference of various center temperatures.

Figure 7a shows the temperature variation at points A, B and C in response to changes in the central chamber temperature. When the heater in the central chamber is activated, heat is primarily concentrated at point A, which lies within the chamber. The temperature at point B, located in the microfluidic channel, is approximately one-third of that at point A. In contrast, the temperature at point C remains nearly unchanged. As shown in Figure 7b, the temperature difference between A and C is greater than that between A and B. This indicates that the suspended bridge structure effectively isolates the central chamber thermally from the surrounding substrate, thereby reducing heat loss and enhancing the thermal performance of the sensor.

From the above analysis, it is evident that this micro-thermal sensor measures temperature change in real time through the reference and main sensors. Compared with the PDMS chambers, the proposed bridge structure with suspended microfluidic channels reduces heat loss and directly improves the sensitivity of thermal detection.

### 3.3. The Response Time Performance of the Device

The response time of the sensor is an essential aspect of this design study as well. A faster response means that the sensor can be activated quickly after the power is applied and quickly enter operation for data acquisition.

**Figure 8:**
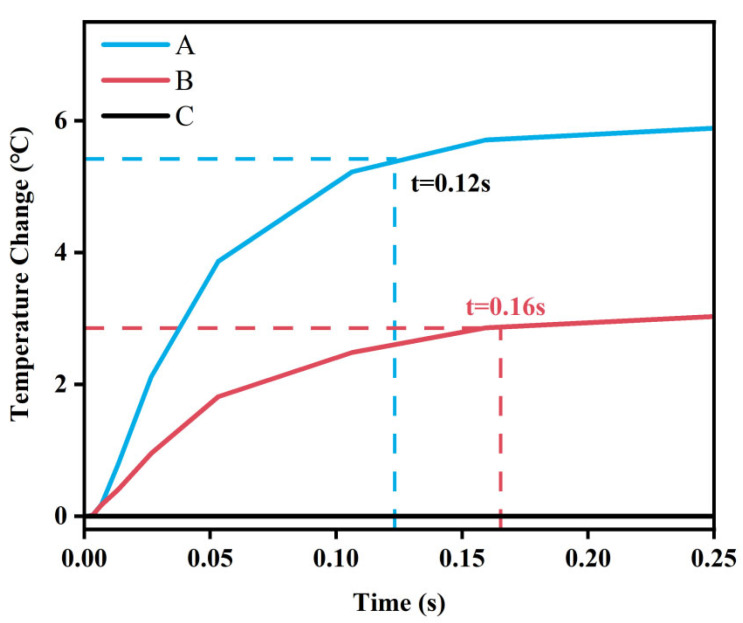
Response time of thermal sensors.

The temperature difference between the main heat sensor (point A) and the reference heat sensor (point B) is converted into an electrical signal for the final output of the sensor. Figure 8 illustrates the temperature variations at points A, B and C during the heating process. Once heating begins, the temperature at the center (point A) rises rapidly. Subsequently, heat is transferred through the microfluidic channel to point B, causing its temperature to increase. After a brief period, the temperatures at points A and B stabilize. This result indicates that the sensor is capable of achieving a millisecond-level rapid response to temperature changes.

### 3.4. Fabrication and Structure of the Device

**Figure 9:**
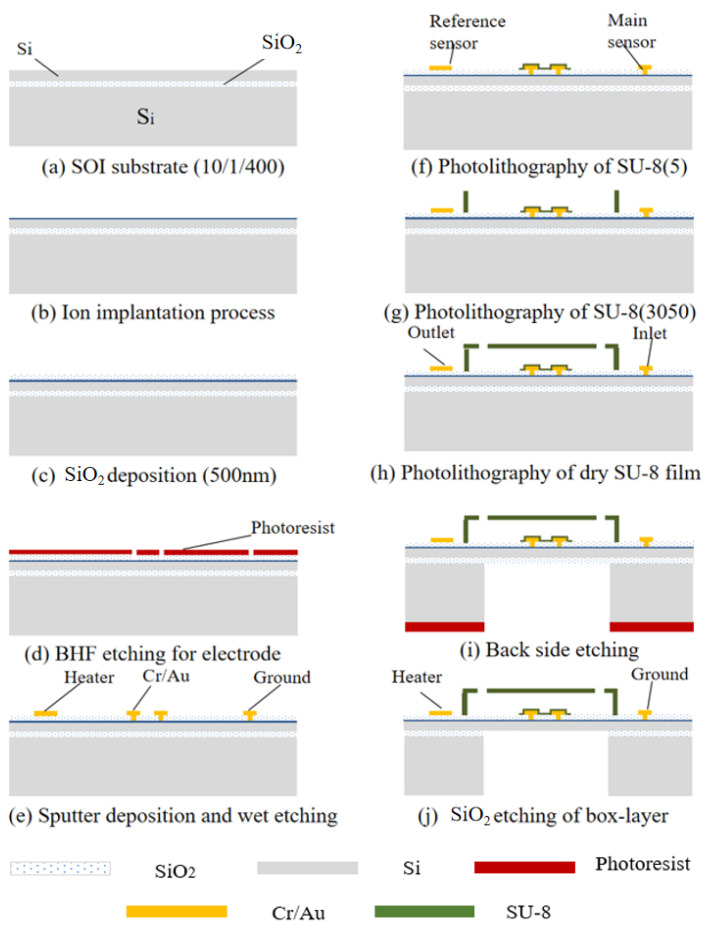
Main processing steps of the fabricated sensor prototype: (**a**) starting with SOI wafers; (**b**) ion implantation process for thermocouple sensor; (**c**) SiO_2_ film deposition by chemical vapor deposition; (**d**) preparation of ports for thermocouple electrodes using a wet etching process; (**e**) sputtering deposition and wet etching process for the electrode of the thermal sensor; (**f**) preparation of separation layers; (**g**) photolithography process for the bottom and wall of the micro-channel; (**h**) fabrication of the cover of the micro-channel with dry SU-8 film; (**i**) backside etching process for the suspended structure; (**j**) backside etching of silicon dioxide.

Figure 9 illustrates the fabrication process of the complete sensor structure, which is based on an SOI substrate. Ion implantation was employed to achieve a low electrical resistivity and high Seebeck coefficient. Subsequently, a thin silicon dioxide film was deposited on the silicon substrate using chemical vapor deposition to serve as an isolation layer. We prepared the ports for the thermocouple electrodes using a wet etching process, and the metallic conductive electrodes were prepared on top of the silicon liner sputter deposition. SU-8 is a common material used to prepare the separation layer. To achieve a robust hollow chamber structure, we fabricated five SU-8 microchannel walls using lithography with rotating coatings and fluidic channels. At the same time, the dry film of SU-8 was covered on the substrate by controlling the force and temperature, and the overlay pattern of the microchannels was fabricated by photolithography. After patterning, microfluidic detection sensors with a bridge structure were prepared by etching the back side of silicon.

**Figure 10:**
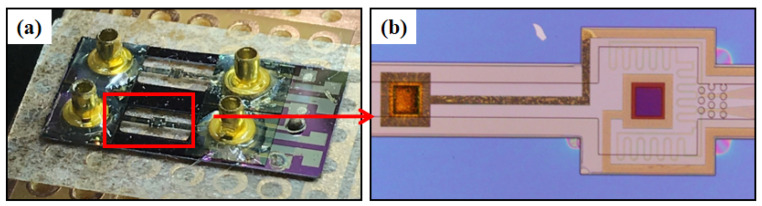
Sensor with suspended bridge structure. (**a**) Photograph of the sensor chip mounted on a printed circuit board. (**b**) Magnified micrograph of a single sensor unit showing the reaction chamber, heater and thermocouples on the suspended microchannel.

Figure 10 shows a schematic diagram of sensors with a suspended bridge structure. The suspended structure was composed of SU-8 dry film. A heater composed of a Cr/Au film surrounded the central chamber. Main sensor and reference sensors were composed of thermocouples set inside the microfluidic channel for thermal detection. In order to evaluate the thermal detection performance of the sensors, experiments were performed using the heaters to simulate the exothermic reaction.

## 4. Results and Discussion

### 4.1. Characterization of Thermal Distribution

**Figure 11:**
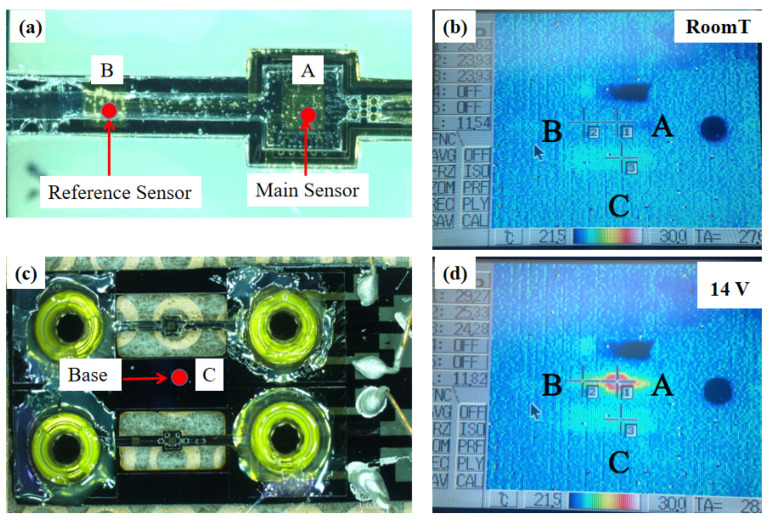
Temperature detection by IR-thermometry. (**a**) Micrograph showing measurement points A and B. (**b**) IR image at ambient temperature before heating. (**c**) Experimental setup showing the location of the substrate measurement point C. (**d**) IR image with the heater activated (14 V), showing significant heat concentration at point A and confirming the structure’s insulating properties.

Figure 11 indicates the temperature distribution measured by a thermal camera at points A, B and C using infrared thermography. The temperature in the initial state was the same. After changing the temperature of the central chamber using a 14 V heater, the temperature change at each point was detected; most of the heat was concentrated at point A, causing the temperature at point A to rise sharply; a small amount of heat was transferred to point B, where the temperature rose slightly; and almost no heat was transferred to point C, where the temperature did not change. This phenomenon proves that the suspension bridge structure is well insulated. The result shows that the suspended bridge structure can reduce heat wastage and improve the detection accuracy of the sensor.

**Figure 12:**
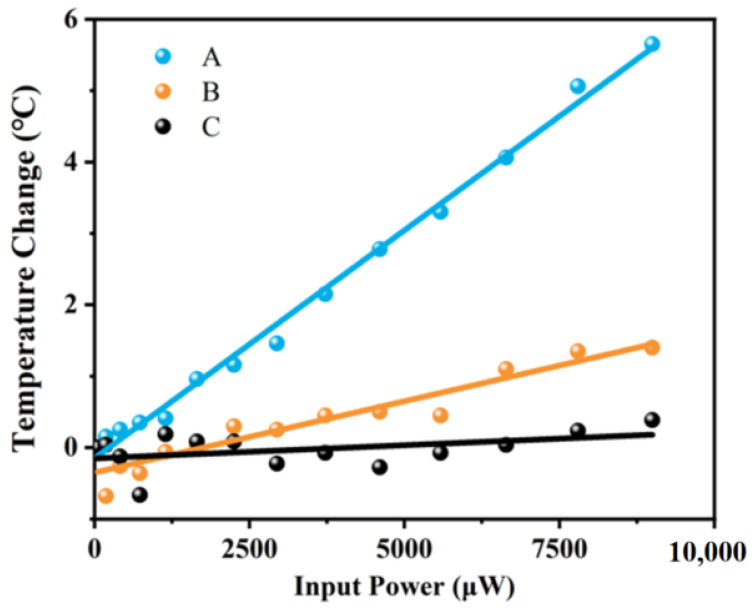
The temperature change of various input powers.

To study thermal behavior, the heater power was varied while monitoring temperature changes at multiple points in the flow channel during sensor operation. The heater was set up around the main sensor as the heat source directly affecting the temperature variation in the central chamber. With increasing heater power, the temperature at point A rises rapidly. Some of the heat was transferred to point B, resulting in a slightly higher temperature at point B than at point C. Due to the excellent thermal insulation of the suspension bridge structure, the temperature at point C remained almost constant and the heat change caused by the heater did not spread to the substrate. When the power of the heater was greater than 2800 μW, the temperature at points A and B increased linearly with heating power. However, no clear linear relationship was observed in the low-temperature region. The temperature change of various input powers are shown in Figure 12.

We used the principle of different degrees of absorption of infrared radiation at different wavelengths to determine its temperature by measuring the monochrome radiation brightness of the object under a certain wavelength. Thermal imager test results can be affected by factors such as test angle and detection distance. The detection data of the sensors in the cryogenic cross-section fluctuates considerably due to the detection instrument.

**Figure 13:**
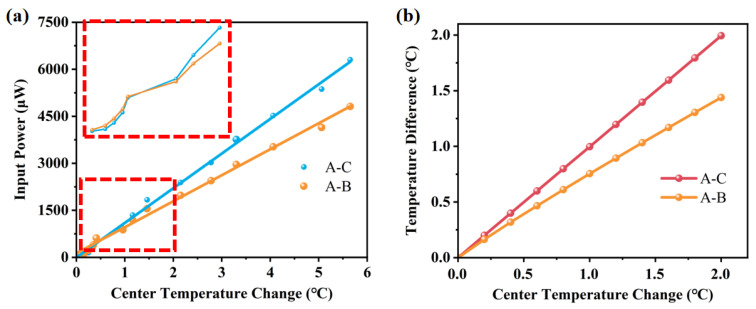
Comparison of experimental and simulated thermal response of the sensor at small temperature differences. (**a**) Temperature variation of the thermal sensor under microheater and temperature change under a tiny temperature difference; (**b**) simulation results under a tiny temperature difference.

Figure 13a shows the temperature changes of the sensor under the microheater. Due to the limitations of the accuracy of the thermal imaging camera, no linear pattern is evident in the temperature variations detected in the low-temperature region. Thus, the data obtained from the actual test is not linear for small temperature variations. Therefore, the temperature change of the sensor in the low-temperature section was simulated. The simulation results confirmed that the sensor itself still has excellent detection performance. Compared with Figure 13a, the simulation results (Figure 13b) are similar to the actual test results, which further confirms the correctness of the simulation.

After analysis, the sensor still maintains fine linearity and accuracy under slight temperature variations. Consequently, the suspension bridge structure designed in this study not only gives good linearity to the sensor, but also greatly reduces the heat loss in the exothermic reactions and improves the accuracy of the sensor. Meanwhile, the simulation results prove that it still has an excellent detection performance at small temperature variations. This property is also important for predicting the test data of sensors in subsequent research design.

### 4.2. Thermal Sensing Performance of the Device

**Figure 14:**
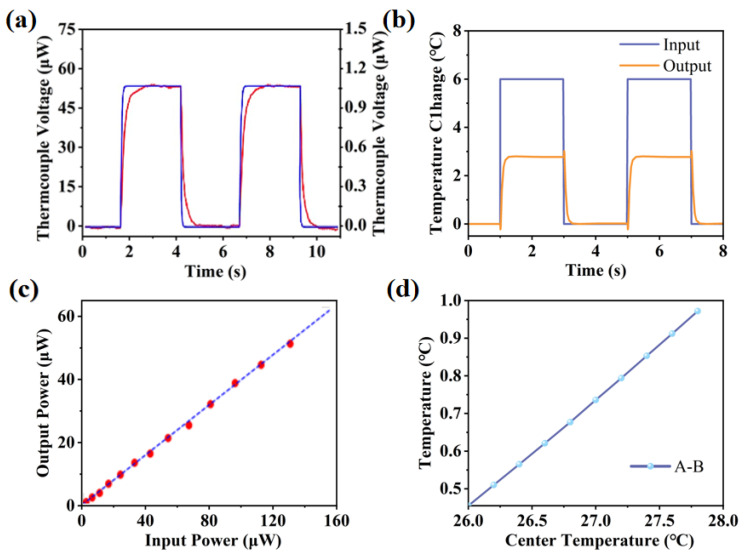
Characterization of the sensor’s thermal performance, comparing experimental measurements with simulation results. (**a**) The fast response of the thermal sensor by a microheater; (**b**) simulation of output temperature variation at different heating temperatures; (**c**) sensor—sensitivity assessment of the sensor by a microheater; (**d**) simulation of temperature variation between A and B at different heating temperatures.

Figure 14a,b indicate the actual test results and simulation results of the sensor in response time. When the step temperature is the input, the sensor is able to respond very quickly to the temperature variation. In less than 200 ms, the thermocouple sensed the temperature change and converted to an electrical output. Furthermore, the output waveform of the sensor followed the same trend as the input waveform, which showed that the actual test results are consistent with the simulation results. The results show that the thermal sensor has a fast response and good thermal properties.

Figure 14c exhibits the results of a sensitivity assessment of the sensor using a microheater. The sensor has a sensitivity of 0.38 V/W and a highly linear relationship between input power and output voltage. The correlation coefficient obtained for the sensor output voltage and input power of the sensor is approximately 1.0, indicating a high accuracy in terms of thermal characterization. Figure 14d shows the sensor sensitivity result obtained from the simulation, where the temperature difference between points A and B is proportional to the input voltage. The result indicates a highly linear relationship between its input and output voltages. The test results are consistent with the actual data, which confirms that the designed thermal sensor can be used for high-precision detection.

### 4.3. Application Prospects of Multi-Component Detection

In previous studies, the design and simulation of a single microfluidic structure verified the superior performance of a suspended microfluidic structure for fluid detection requirements. In order to enable multifunctional detection and independent temperature detection within each chamber, multiple microfluidic structures are designed in this paper for application in multi-target detection.

**Figure 15:**
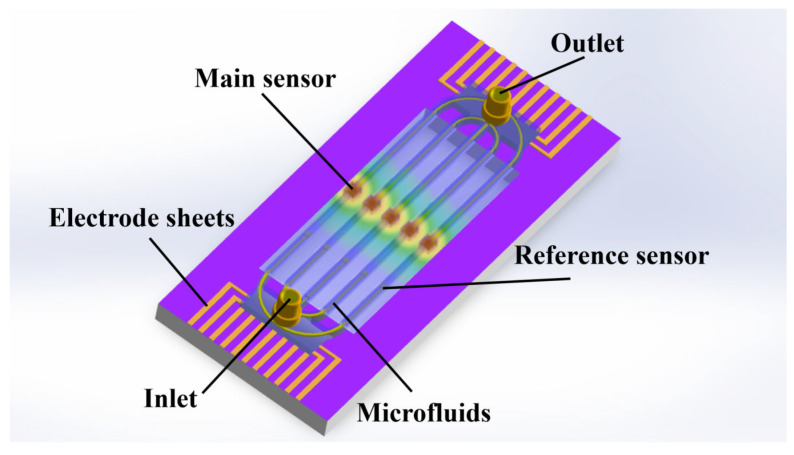
Integral structure of the detection sensor.

Figure 15 shows the design of the multifluidic suspension structure. For simulating different thermal reaction processes, different temperatures were set within each fluidic channel, from left to right, at 28 °C, 30 °C, 32 °C, 30 °C and 28 °C, respectively. The simulation results show that the temperature distribution within each flow channel is similar to that of the individual flow channels and that each flow channel does not affect the others. Therefore, the multifluidic channel sensor is fully capable of multi-target detection.

Multiple microfluidic structures were applied to the multi-component detection for liquid measurements. When the sensor starts operating, the solution to be measured is first injected by titration into the detection chamber, which fills the entire flow channel. The solution generates a different thermal reaction in the microfluidic channel. Finally, the heat of the reaction can be detected by the micro-thermal sensor for multi-component analysis.

The cost-effectiveness of the proposed thermal silicon-based microfluidic sensor primarily stems from two aspects:

Simplified MEMS Fabrication: The sensors utilize standardized silicon-on-insulator (SOI) wafers and commercially available SU-8 photoresist (Shin-Etsu, MicroChem), avoiding expensive materials like noble metals or complex multilayer structures. The single-mask process for microchannel formation and batch fabrication (6-inch wafers yielding > 200 chips) significantly reduces unit costs.

Scalable Manufacturing: The suspended microfluidic structure is fabricated via conventional MEMS processes (e.g., photolithography, dry etching) without the need for specialized equipment. Compared with PDMS-based microfluidics requiring soft lithography and plasma bonding, our SU-8 monolithic integration eliminates assembly steps, cutting labor and material waste.

While exact unit costs depend on volume, preliminary estimates suggest < USD 5 per chip at scale, based on material costs (USD 1.2/wafer for SOI, USD 0.8 for SU-8) and shared facility usage fees. This is competitive against commercial electrochemical urine sensors (e.g., USD 20–USD 50/test strip).

## 5. Conclusions

In this study, an MEMS-based thermal sensor featuring a novel suspended bridge microfluidic structure was successfully designed, fabricated and characterized. By utilizing SU-8 material and MEMS technology, this design overcomes the process constraints of conventional PDMS-based devices and significantly improves thermal management. Theoretical calculations, validated by experimental results, confirm that the suspended structure reduces heat loss by 88.64% compared with non-suspended designs. The resulting sensor demonstrates excellent performance, including an ultra-high sensitivity of 0.38 V/W, a rapid response time of less than 200 ms and a strong linear correlation (R^2^ ≈ 1.0) between input power and output signal.

The significance of this work extends beyond its performance metrics. The proposed fabrication method is fully compatible with standard MEMS processes, indicating a strong potential for cost-effective and scalable mass production. Furthermore, the single-channel architecture has been shown to be a stable and reliable platform, which can be readily expanded into multi-channel arrays. This scalability is crucial for developing integrated biosensors capable of simultaneous, multi-component analysis, thereby advancing the development of portable, high-efficiency point-of-care testing (POCT) systems for complex biological samples.

This sensor architecture holds considerable promise for multifunctional biomedical detection, and future work will focus on translating this potential into practical applications. Key next steps include: (1) conducting large-scale clinical validation with real-world samples to assess diagnostic accuracy; (2) developing multi-channel arrays functionalized with target-specific enzymes for the simultaneous detection of diverse biomarkers (e.g., glucose, urea); and (3) optimizing enzyme immobilization protocols. These advancements will accelerate the creation of low-cost, home-based diagnostic devices for chronic disease monitoring and management.

## Data Availability

Data is contained within the article; The original contributions presented in this study are include in the article. Further inquires can be directed to the corresponding author.
